# Characterization and Cytotoxic Activity of Microwave-Assisted Extracted Crude Fucoidans from Different Brown Seaweeds

**DOI:** 10.3390/md21010048

**Published:** 2023-01-11

**Authors:** Ahmed Zayed, Doris Finkelmeier, Thomas Hahn, Lisa Rebers, Anusriha Shanmugam, Anke Burger-Kentischer, Roland Ulber

**Affiliations:** 1 Institute of Bioprocess Engineering, Rheinland-Pfälzische Technische Universität Kaiserslautern-Landau, Gottlieb-Daimler-Straße 49, 67663 Kaiserslautern, Germany; 2 Department of Pharmacognosy, College of Pharmacy, Tanta University, Tanta 31527, Egypt; 3 Innovation Field Cell and Tissue Technologies, Fraunhofer Institute for Interfacial Engineering and Biotechnology IGB, Nobelstraße 12, 70569 Stuttgart, Germany; 4 Innovation Field Industrial Biotechnology, Fraunhofer Institute for Interfacial Engineering and Biotechnology IGB, Nobelstraße 12, 70569 Stuttgart, Germany; 5 Biology Department, Rheinland-Pfälzische Technische Universität Kaiserslautern-Landau, Erwin-Schrödinger-Straße 13, 67663 Kaiserslautern, Germany

**Keywords:** anti-tumor, fucoidans, extraction, microwave-assisted, bioactivity, brown algae

## Abstract

Microwave-assisted extraction (MAE) is recognized as a green method for extraction of natural products. The current research aimed to explore the MAE for fucoidans extraction from different brown seaweeds, including *Fucus vesiculosus*, *F. spiralis*, and *Laminaria saccharina*. Following several solvent-extraction pre-treatment steps and MAE optimization, the algal biomasses were extracted in a ratio of 1:25 in 0.1 M HCl containing 2 M CaCl_2_ for 1.0 min. The results showed that *L. saccharina*’s extract was different from the others, regarding the highest sugar content reached 0.47 mg glucose equivalent/mg extract being confirmed by monosaccharide composition analysis and the lowest fucoidan content and sulfation degree at 0.09 mg/mg extract and 0.13, respectively. Moreover, these findings were confirmed by tentative structural elucidation based on Fourier-transform infrared spectrometry which also showed a different spectrum. However, the MAE enhanced melanoidins formation in products, which was confirmed by the intense band at 1420 cm^−1^. Interestingly, the results of monomeric composition showed that fucoidan extract by MAE from *F. vesiculosus* belonged to sulfated galactofucans which are known for their potential bioactivities. Furthermore, the cytotoxic activity of the four fucoidans in concentrations ranging from 4.9 µg/mL to 2500 µg/mL was investigated and correlated with the chemical characterization showing that *F. vesiculosus*_MAE fucoidan was the most potent and safest. The current research revealed the chemical heterogeneity of fucoidans regarding taxonomical class and used greener extraction method of fucoidans toward the achievement of the UN sustainability goals.

## 1. Introduction

Brown algae are diverse marine organisms with more than 480 species that contain wide array of potential bioactive secondary metabolites, e.g., sulfated polysaccharides (fucoidans), fucoxanthins, alginic acid, laminarin, and phlorotannins [1,2]. Specifically, fucoidans are a heterogenous class of sulfated, water-soluble, fucose-rich polysaccharide exhibiting unique chemical properties and various health promoting benefits, and consequently find many applications in different fields of cosmetics, drug delivery, and therapeutics [3,4]. Hence, different downstream processes have been optimized for the recovery of fucoidans with improved yield and in a high-quality grade devoid of other co-extracted contaminants [2,5].

*Fucales* are known to be rich in fucoidans, which are used for production of fucoidans commercially, especially *Fucus vesiculosus* [2]. However, little bit of information is available in previous literature regarding fucoidan isolated from other species as *Fucus spiralis* with respect to extraction, chemistry, and bioactivities, compared to *F. vesiculosus* and *F. serratus* [6,7,8]. Most of bioactivities of *F. spiralis* have been documented to its phlorotannins containing fractions, which possessed potential antioxidant and cytoprotective activities [9,10]. Additionally, *Laminaria saccharina* (syn. *Saccharina latissima*) has been investigated for its fucoidan content in previous publications. Its fucoidan backbone was reported to be built up of (1→3) linked *α*-_L_-fucopyranose monomers which are sulfated mainly at C-4. Nevertheless, additional C-2 sulfation and 2-*O*-*α*-_L_-fucopyranosyl branching were also documented [11].

Fucoidans have shown potential bioactivities, including antioxidant, anti-coagulant, anti-tumor, anti-inflammatory, and management of metabolic diseases and kidney dysfunction [12,13,14,15,16,17,18,19] with furtherly formulated in pharmaceutical preparations as an essential step for clinical investigations and commercial marketing [20]. However, the recent studies found that the antioxidant activity is correlated potentially with the phenolic contents in crude fucoidan extracts [21]. Interestingly, fucoidans’ chemical and physico-chemical properties have been successfully related with their bioactivities in numerous structure-activity relationship studies such as sulfation degree and molecular weight, and monomeric composition [12,21,22]. Particularly, the antitumor activity of fucoidan fractions is typically attributed to low molecular weight and high sulfation degree [23,24]. Asides, the mechanism of action has been explained by induction of apoptosis and cell cycle arrest on the cellular level [25]. For instance, the breast adenocarcinoma MCF7 cell line showed that apoptosis can be induced via reactive oxygen species (ROS)-dependent JNK activation and mitochondria-mediated pathways due to fucoidan treatment [26], e.g., via caspase-8 [27] and caspase-9 activation [28]. For colorectal adenocarcinoma Caco-2 cell line opposing phenomena were observed after incubation with fucoidan fractions: on the one hand, apoptotic effects were observed due to enhanced ROS production [29] and on the other hand protective effects against hydrogen peroxide by enhanced intestinal epithelial barrier function were found [30].

Biorefining of macroalgae metabolites is a relatively recent approach with potential industrial applications such as in pharmaceuticals and cosmeceuticals [31]. As a potential part of fucoidan downstream processes, extraction methods can be classified into classical solvent and modern methods. These methods have been previously reviewed addressing their potential applications regarding various factors as product yield and quality [2]. Particularly, modern extraction methods (e.g., supercritical fluid extraction (SFE), ultrasound-assisted extraction (UAE), enzyme-assisted extraction (EAE), and microwave-assisted extraction (MAE)) are characterized by short extraction times, high production yield, and use of small extraction volumes and non-corrosive solvents, compared to conventional solvent extraction counterpart [2,32]. Especially, MAE has acquired a great interest in the field of natural products recovery from seaweeds, specifically sulfated polysaccharides as fucoidans and phlorotannins from brown seaweeds [33,34], in addition to ulvan from green seaweeds [35]. This kind of extraction is based on the electromagnetic waves resulting in a distributed heat source that enhance cell wall degradation and release of fucoidan into the aqueous extraction solvent [36].

To the best of our knowledge, MAE has been applied for fucoidans extraction from small number of brown seaweeds, as in case of *Fucus vesiculosus* [33], *Fucus virsoides* and *Cystoseira barbata* [37], and *Ascophyllum nodosum* [38]. However, it has not been reported for *F. spiralis* and *L. saccharina*, which have been extracted by classical solvent extraction containing CaCl_2_ followed by ethanol precipitation [8,39]. The results of MAE experiments showed improved yields, cost-effectiveness, less use of solvents, and energy and time saving benefits, compared with the classical solvent extraction method. Hence, these benefits are in agreement with and help achievement of the United Nations Sustainable Development Goals (SDGs) [40].

Therefore, the current research aimed at investigating MAE of three brown algae, i.e., *F. vesiculosus*, *F. spiralis*, and *L. saccharina*. *F. spiralis* and *L. saccharina* were chosen since there have been not enough data regarding this extraction method, while *F. vesiculosus* for comparison with our previous conventional solvent extraction method [41,42]. In addition, the selective cytotoxic activity of these four fucoidan extracts was investigated against primary fibroblasts from healthy tissue and two cell lines MCF7 and Caco-2 from adenocarcinomas. The use of these cells enabled the comparison of cytotoxic activity against cells from healthy and cancerous tissues.

## 2. Results and Discussion

### 2.1. Pre-Treatment and Extraction Optimization

Fucoidan makes up only 12.4% of the biomass of *F. vesiculosus* alga on average for instance, making it a minor component of the biomass in comparison to other substances, including other carbohydrates [43]. Hence, pre-treating the algae biomass is beneficial to avoid the co-extraction of other algal components during the separation of fucoidan. Pre-treatment is therefore frequently used to get rid of phenols, pigments, and lipids as possible [41,44]. The pre-treatment protocol consisted of various solvent extractions, which was performed to remove co-extracted contaminants, including pigments, protein, and phenolic compounds [41]. Then, a combination of acidulated water containing CaCl_2_ was applied to improve fucoidan’s yield, in addition to precipitate the alginate as Ca salt. Furthermore, the extracts were neutralized directly after extraction step by ammonium carbonate, which is mild alkali preventing the excessive acid and alkali hydrolyses of polymer and sulfate ester groups, besides to assure the precipitation of excess CaCl_2_ as CaCO_3_.

According to numerous investigations performed previously, MAE can create biopolymers at heating time that are much lower than those required by traditional extraction techniques [45]. In the current investigation, MAE was firstly employed to optimize fucoidan extraction from *F. vesiculosus*, where the best condition could be used afterwards in further extraction upscaling for the other algal species. Power, extraction time, and solid/liquid ratio were selected based on previous studies for the production of fucoidan from *F. vesiculosus* [33]. The results of this step are summarized in Table 1.

The results in Table 1 showed for extraction yield that the solid/liquid ratio (1 g/25 mL) of conditions 1, 2, and 5 was superior to 1 g/10 mL of condition 3 and 4. In addition, the extraction time had not shown potential main effect for extraction yield at condition 2. Asides, decreasing of extraction power to 30% (240 W) in condition 5 resulted in increasing of the extraction yield to 12.25% *w*/*w* in agreement with previous reports [46]. However, the chemical characterization of fucoidan extracted from *F. vesiculosus* at condition 1 revealed higher fucoidan, sugar, and sulfate contents at 0.64, 0.18, and 0.092 mg/mg extract, compared to condition 5. Hence, the extract quality of condition 1 was better and consistent with previous experiments performed by Rodriguez-Jasso et al. [33]. These findings encouraged us to use condition 1 for the further upscaling extraction of fucoidans from the other algae, including *L. saccharina*, *F. spiralis*, and *F. vesiculosus* as well.

Previously, classical solvent extraction showed that 24.5% *w*/*w* were derived from pre-treated *F. vesiculosus* with fucoidan content of 0.73 mg/mg extract [47,48]. This result indicated that the classical solvent extraction method was more efficient for fucoidan extraction from *F. vesiculosus*. However, the classical method was performed for longer time for 3 h, twice extraction, and use of 1:10 biomass to solvent ratio.

### 2.2. Chemical Characterization

#### 2.2.1. Fucoidans-Related Contents

Diverse characters of the extracted fucoidans were investigated to assess the applied extraction technique, extracted products, and to give a nearly complete chemical picture before the following bioactivity assays. The results are summarized in Table 2 regarding sugar content, fucoidan content, sulfation degree, total phenolic content (TPC), and protein contents.

The results in Table 2 showed that fucoidan isolated from *L. saccharina* was relatively different from other fucoidans with respect to all investigated parameters. It showed the highest sugar content with 0.47 mg glucose equivalent/mg extract compared with 0.12 and 0.13 mg glucose equivalent/mg extract for *F. spiralis* and *F. vesiculosus*, respectively, extracted by the same conditions, whereas *F. vesiculosus* extracted by classical solvent extraction also showed lower sugar content [47].

Additionally, determination of fucoidan content by toluidine blue assay used *F. vesiculosus* and *Undaria pinnatifida* as reference standards since there is no reference fucoidan derived from *L. saccharina* or *F. spiralis* commercially. The results showed that *L. saccharina* contained low fucoidan content with only 0.09 and 0.11 mg/mg extract equivalent to fucoidan derived from *F. vesiculosus* and *U. pinnatifida*, respectively. However, the other fucoidans type showed comparable content. Nevertheless, the classical extraction showed higher content compared with MAE for *F. vesiculosus* fucoidan. Such findings were confirmed by the elemental analysis and calculations of sulfation degree, where *L. saccharina* showed a degree of sulfation of 0.13, while the other *Fucus* sp. showed nearly similar sulfation degree around 0.74 near to that extracted by solvent extraction. These results are in agreement with previous reports, which showed that *L. saccharina* contained a mannoglucuronofucans fraction, which is partially sulfated, i.e., fraction L.s.-1.0 [49].

Moreover, Mw measurement revealed similar values of MAE fucoidans at 16.53, 15.87, and 16.42 kDa for *F. vesiculosus*_MAE, *F. spiralis*_MAE, and *L. saccharina*_MAE, which are considered lower than reported previously for *F. vesiculosus* by classical solvent extraction at 64 kDa [42]. The decrease obtained Mw of MAE fucoidans indicated a high possibility of polymer hydrolysis in comparison with that extracted by classical solvent extraction. Moreover, MAE is considered a more drastic extraction method compared with EAE, which was used with *L. saccharina* and resulted in fucoidan with Mw of 50–100 kDa [50].

Furthermore, the chromatograms of monosaccharide composition analysis shown in Figure S1 (Supplementary Material) resulted in identification of galactose and glucose in all fucoidans, in addition to glucose which was not found in *F. vesiculosus*_MAE. The ratios between glucose:galactose:fucose were 0.0:0.03:1.0, 0.03:0.03:1.0, 0.92:0.09:1.0, and 0.02:0.03:1.0 in *F. vesiculosus*_MAE, *F. spiralis*_MAE, and *L. saccharina*_MAE, and *F. vesiculosus*_solvent extraction (SE), respectively. The high ratio of glucose in *L. saccharina*_MAE confirmed the high sugar content shown in Table 2. Additionally, the absence of glucose in *F. vesiculosus*_MAE indicated its galactofucan nature and might propose a superior bioactivity compared with the other fucoidans of the current study [22].

#### 2.2.2. Co-Extracted Contaminant Contents

Particularly, protein and phenolic contents were investigated to assure the selectivity of applied extraction techniques. Additionally, the contamination of fucoidan extracts with polyphenolic components might results in false bioactivity results [51]. Hence, the ethanol:water:formaldehyde (80:15:5, pH 2.0) step in pre-treatment protocol was included, since this condition could polymerize phenolic compounds, enhancing their removal in the consequent step of 80% ethanol [2]. The results showed that this step was successful in removal of both potential contaminants, except that of *F. vesiculosus*-MAE, which still contained minute traces of TPC of 1.64 µg GAE/mg extract, Table 2. Comparison with previous reports that extracted fucoidans without pre-treatment steps also confirmed the usefulness of this extraction protocol. For instance, the phenolic content, i.e., phlorotannins and flavonoids, in *F. vesiculosus* that has been recently determined by Obluchinskaya et al. was at 72.4–158.1 mg phloroglucinol equivalent/g dried weight (DW) algae based on geographic locations of algae harvesting [52]. Moreover, the phenolic and protein contents in other species of brown seaweeds as *Colpomenia sinuosa* were at 0.045 mg GAE/g DW and 1.88%, respectively [53].

#### 2.2.3. Structural Features

**Figure 1:**
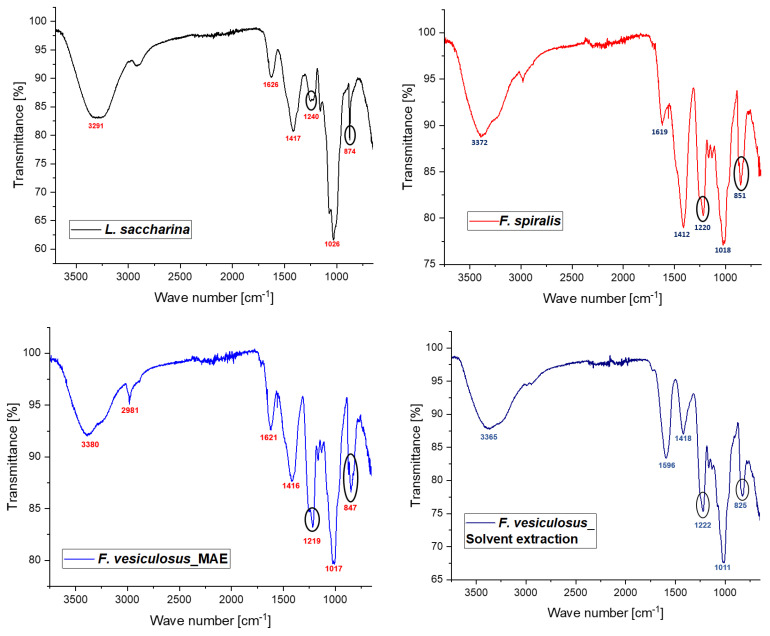
FT-IR spectra of fucoidans extracted from various brown seaweeds by MAE in comparison with *F. vesiculosus* fucoidan extracted by solvent extraction method.

Fourier-transform infrared spectrometry coupled with attenuated total reflectance (ATR-FT-IR) was employed to distinguish the characteristic functional groups in extracted fucoidans, and in comparison with *F. vesiculosus* from Sigma-Aldrich (>95% pure). Intact sulfate groups should provide bands at approximately 1220 cm^−1^ and 840 cm^−1^ for asymmetric stretching vibration of S=O and C-O-S, respectively. In addition, the complex IR pattern around 833 cm^−1^ and appearance of a shoulder at 815 cm^−1^ may indicate the of equatorial substitution of sulfate ester group at C-4 position [41]. The IR spectra in Figure 1 confirmed the previous findings and showed that *L. saccharina* fucoidan is atypical, where the S=O band at 1240 cm^−1^ was wide and compressed, in addition to the C-O-S band at 874 cm^−1^ was reduced to a narrow band without showing any complicated pattern. Nevertheless, fucoidans derived from *Fucales*, i.e., *F. spiralis* and *F. vesiculosus* showed relatively similar IR spectra revealing the complicated region at 840 cm^−1^ being suggestive for the equatorial pattern of sulfation at C-4 and C-2 consistent with previous publications [41,54]. Though, with comparison with the purified form of *F. vesiculosus*, all extracted fucoidans showed an additional band at around 1420 cm^−1^, which may correspond to -C-H deformation of formed melanoidins [54]. The higher intensity of this band in MAE products suggested that the higher temperature accompanied with MAE helped in Maillard reaction occurrence between protein and carbohydrate contents resulting in formation of melanoidins. Yet, further structure elucidation based on ^1^H- and ^13^C-NMR is highly recommended to fully characterize the produced fucoidans.

### 2.3. Evaluation of Cytotoxic Activity

**Figure 2:**
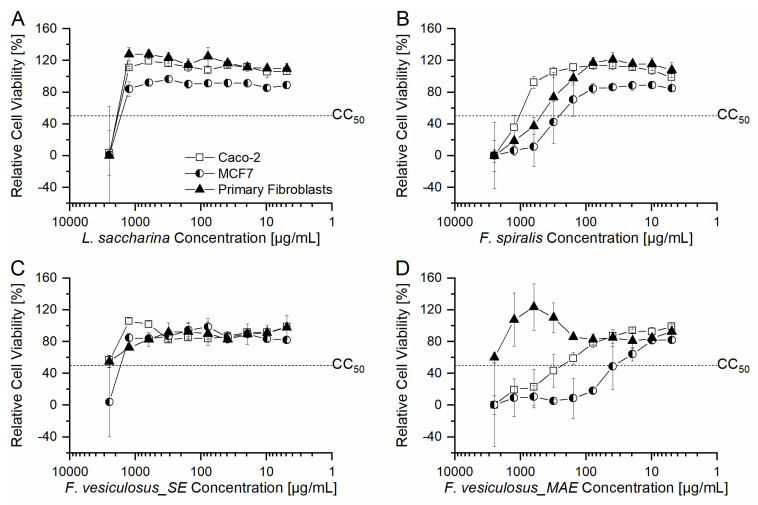
Dose–response curves of primary fibroblasts (triangles), MCF7 cell line (squares) and Caco-2 cell line (circles) treated with four different fucoidan fractions in concentrations ranging from 4.9 µg/mL to 2500 µg/mL: *L. saccharina* (**A**), *F. spiralis* (**B**), *F. vesiculosus*_solvent extraction (SE) (**C**), *F. vesiculosus*_MAE (**D**). The relative cell viability was calculated relative to the untreated negative control. Furthermore, the half-maximal cytotoxicity (CC_50_) was drawn in for better guidance of the eye. The legend shown in (**A**) is representative for all diagrams.

The viability of cells treated with fucoidan fractions in concentrations ranging from 4.9 µg/mL to 2500 µg/mL was determined by the enzymatic conversion of non-fluorescent FDA to fluorescent fluorescein by cellular esterases. Released fluorescein was measured by fluorescence measurements and was put in relation to the negative control (100% relative cell viability) resulting in the relative cell viability. The dose–response curves of the three different cell types treated with four different fucoidan fractions are shown in Figure 2, relative viabilities of cells treated with control substances are given in Figure S2 in the Supporting Information. Another data presentation sorted according to cell types is given in Figure S3 in the Supporting Information. Furthermore, the half-maximal cytotoxic concentrations (CC_50_) were calculated out of the dose–response curves shown in Figure 2, these CC_50_ values are given in Table 3. The statistical evaluation of CC_50_ values is given in the Supporting Information (Supplementary Material, Tables S1 and S2).

The general pattern of the dose–response curves of primary fibroblasts, MCF7 and Caco-2 cell lines treated with the fucoidan fractions *L. saccharina*, *F. spiralis* and *F vesiculosus*_SE were similar (see Figure 2A–C), whereby just the highest concentration (2500 µg/mL) of *L. saccharina* and *F. vesiculosus*_SE led to relative cell viabilities < 50%. Furthermore, the CC_50_ of Caco-2 and primary fibroblasts treated with *L. saccharina* were similar (*p* > 0.05, see Table S2). For *F. spiralis* concentrations higher than 625 µg/mL (Caco-2), 312.5 µg/mL (primary fibroblasts) and 156.25 µg/mL led to relative cell viabilities < 50%, respectively, whereby the CC_50_ values differed significantly between the cell types (*p* < 0.05, see Table S2). The fucoidan fraction *F. vesiculosus*_MAE (Figure 2D) showed cell type-dependent differences in the dose–response curves: Primary fibroblasts were affected less and showed relative cell viabilities > 50% for all investigated concentrations, while the relative cell viability of the two cancerous cell lines were most cytotoxically affected compared with the other three fucoidan fractions.

**Figure 3:**
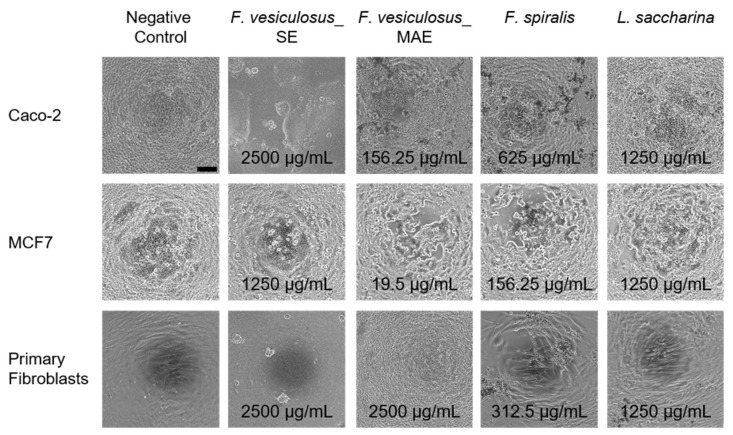
Representative phase contrast images of different cell types (Caco-2 (upper row), MCF7 (middle row), primary fibroblasts (lower row) after incubation with different fucoidan fractions in a concentration that was lower than but as close as possible to the CC_50_, specified in each individual image. Furthermore, the left column shows the negative controls. The scale bar of the image in the left corner represents 100 µm and is representative for all images.

In addition to the investigation of relative cell viability, the morphology of the three different cell types (primary fibroblasts, MCF7, and Caco-2 cells) after treatment with the four fucoidan fractions was analyzed using phase contrast images. Figure 3 shows representative phase contrast images of the three cell types cultivated with the four fucoidan fractions in a concentration that was lower than but as close as possible to the CC_50_ (see Table 3) and representative images of cells cultivated with cell-specific media (negative control). Further phase contrast images of cells treated with controls are shown in Figure S4 in the Supporting Information.

Cells treated with cell-specific media instead of fucoidan fractions (Figure 3, left column, negative control) presented themselves as homogeneous and consistent cell layer. In general, the incubation with different fucoidan fractions lead to contraction within the cell monolayer up to clumping and agglomeration of the cells with increasing fucoidan concentration. It was demonstrated before that treatment with different fucoidan fractions led to shrunk and rounded cells [42,55,56,57]. This morphology change was accompanied by the accumulation of F-actin in the cell cortex, which probably resulted in the round cell morphology [55,56]. Furthermore, these observations were made for fucoidan fractions higher than 100 µg/mL, which is in congruence with the results presented in this study. Comparing the dose–response curves and the calculated CC_50_ values (Table 3) of the three investigated cell types, it stands out that MCF7 cells were most sensitive to tested fucoidan fractions, since the respective CC_50_ values of all fucoidan fractions were the lowest. This observation was consistent with our previous studies investigating the cytotoxic activity of other fucoidan fractions on primary fibroblasts, MCF7 and Caco-2 cells [42], whereas the CC_50_ values of primary fibroblasts were highest for *F. vesiculosus*_MAE and *L. saccharina*. The CC_50_ values of primary fibroblasts and Caco-2 cells treated with *F. vesiculosus*_SE were outside the concentration range investigated in this study. Furthermore, Caco-2 cells showed the lowest cytotoxic effect and thus highest CC_50_ value after treatment with *F. spiralis* fucoidan compared with the other two cell types investigated in this study.

The results of the cytotoxic activity of different fucoidan fractions in this study were consistent with studies by others that have already shown that fucoidans from brown algae exhibited broad biological activity against breast and colon carcinoma cell lines. For example, Cumashi et al. showed that 100 µg/mL fucoidan extracted from *L. saccharina* and *F. vesiculosus* reduced the adhesion of the highly metastatic breast cancer cell line MDA-MB-231 to platelets by ~80%. However, it was shown in the same study that fucoidans from *F. spiralis* did not inhibit the cell adhesion to platelets, but instead inhibited angiogenesis in vitro [11]. The cytotoxic activity of fucoidans extracted from *F. vesiculosus* by induction of apoptosis was shown for colon carcinoma cell line, e.g., HCT-15 [58], HCT-116 [59] and HT-29 [60], and breast cancer cell line, e.g., MCF7 [28] and 4T1 [61,62].

In our previous study, a correlation between the sulfation degree of the fucoidan fractions and the cytotoxic activity was found, whereby a high sulfation degree led to a low CC_50_ value [42]. This context was found in this study too, since *L. saccharina* fucoidan had the lowest sulfation degree (0.13) and lead to high CC_50_ values in all three cell types (Table 3, lower row). High sulfation degrees and corresponding low CC_50_ values were found in the two fucoidan fractions *F. spiralis* (0.73) and *F. vesiculosus*_MAE (0.75), except for Caco-2 and primary fibroblasts, respectively. A direct correlation between molecular weight and cytotoxic activity was not found in this study.

However, the sulfation degree of *F. vesiculosus*_SE (0.67) was in the same range as that of *F. vesiculosus*_MAE and *F. spiralis*, but lead to a significantly lower cytotoxic effect, pointing out that the sulfation degree is just one parameter, which determines the cytotoxic activity of fucoidan fractions, as already observed by others [63]. It was reported that the position of the sulfate ester groups determines their biological activity, whereby sulfate ester groups at C-4 position showed the highest cytotoxicity [28,64]. Zhurishkina et al. reported that fucoidan fractions from *F. vesiculosus* owning sulfate groups predominantly in C-4 position, low uronic acid content and enhanced fucose quantity were most cytotoxic against HeLa-63 and Hep G2 cells [63].

Of particular interest were the differences in the dose–response curve of the two *F. vesiculosus* fucoidans due to their extraction process (solvent extraction vs. MAE): CC_50_ values of malignant cell lines Caco-2 and MCF7 were high after treatment with solvent extracted *F. vesiculosus* fucoidan (>2500 µg/mL and 1786.1 µg/mL), whereas the respective CC_50_ values were low after treatment with microwave-assisted extracted *F. vesiculosus* fucoidan (244.9 µg/mL and 37.6 µg/mL). Yet, the cytotoxic activity of *F. vesiculosus* fucoidan fractions against primary fibroblasts was not affected by the extraction process; CC_50_ were high (>2500 µg/mL) for both fucoidan fractions. The chemical characterization of the fucoidan fractions (Table 2) shows that the main difference between the solvent extracted and microwave-assisted extracted *F. vesiculosus* fucoidan fraction was the high phenolic content of the MAE fraction. Phenolic compounds are known to show specific activity against cancer and tumors [65,66]. Additionally, the monosaccharide composition indicated that the *F. vesiculosus*_MAE fucoidan belongs to the sulfated galactofucans which are characterized by their outstanding and potential bioactivities [22]. Hence, the bioactivity of phenolic residuals and sulfated galactofucan skeleton might explain the higher cytotoxic behavior against MCF7 and Caco-2 cell lines of *F. vesiculosus*_MAE compared with the other fucoidan fractions.

## 3. Material and Methods

### 3.1. Algae Harvesting and Drying

**Figure 4:**
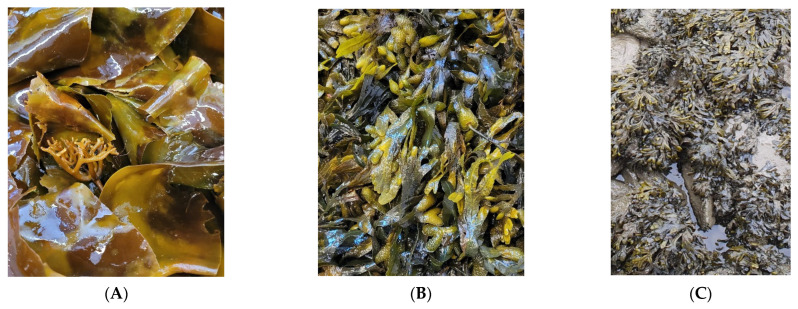
Harvested brown algae of the current study. (**A**) *Laminaria saccharina*, (**B**) *Fucus spiralis*, and (**C**) *F. vesiculosus*.

In early summer 2022, the algal biomasses were harvested where they possessed mature receptacles and in the stage of gametes release according to previous literature [52,67]. *F. vesiculosus* (Voucher sample ID: FV_1001_WH) was harvested and identified by Prof. Dr. Roland Ulber from the south beaches of the North Sea at Wilhemshaven, Germany, while *F. spiralis* (Voucher sample ID: FS_1001_AW), and *L. saccharina* (Voucher sample ID: LS_1001_AW) by Alfred Wegener Institute, Helmholtz Center for Polar and Marine Research (Bremerhaven, Germany), Figure 4. The samples were washed by seawater directly after harvesting and transferred within three days to the lab in Kaiserslautern in ice boxes. As soon as the samples arrived at the lab, they were washed several times under a stream of tap water to remove co-collected contaminants, including sand, dirt, and symbiotic organisms.

The samples were air-dried first at room temperature for one week, and then in the oven adjusted at 50 °C for four days. Afterwards, the dried algae were milled producing their corresponding powders, which were stored in well-tight plastic containers at room temperature until further pre-treatment and extraction processes. Additionally, all voucher specimens were archived at the institute of Bioprocess Engineering, Technical University of Kaiserslautern, Kaiserslautern, Germany.

### 3.2. Pre-Treatment Protocol

The pre-treatment protocol was carried for 100 g of each algae powder following our developed method with few modifications [41]. In brief, acetone, hexane:isopropanol (3:2), 80% ethanol, ethanol:water:formaldehyde (80:15:5, pH 2), 80% ethanol were five pre-treatment stages performed in shaker incubator at 30 °C, adjusted at a shaking rate of 120 rpm, for 24 h, and in a powder to solvent ratio of 1:10, except for acetone which was only for 16 h and in a ratio of 1:15. After each step, the suspensions were filtered through Whatman filter paper, and the filtrates were decanted. The final produced powders were dried in the oven at 55 °C for few days until giving constant weights.

### 3.3. Extraction

A laboratory microwave (MW 4000 HLL Landgraf laboratory system, Langenhagen, Germany) with a maximum power of 800 W and capacity of 220–240 V, 50/60 Hz was employed for algae extraction in combination with stirring adjusted at 800–1000 rpm. According to Rodriguez-Jasso et al., MAE was conducted for fucoidan extraction from *F. vesiculosus* using various parameters [33], where different biomass to solvent ratio, power %, and time were applied as shown in Table 4.

Based on the results of this step, condition 1 was shown to be applied for the further fucoidans extraction. Hence, the three pre-treated algae were extracted for 1.0 min and in a ratio of (1:25, 2.0 g pre-treated algae biomass/50 mL) in duplicates. However, 0.1 M HCl containing 2 M CaCl_2_ was used to enhance the extraction of fucoidan and precipitation of alginate. Then, the extraction containers were left at room temperature for cooling before filtration, where the filtrates were neutralized to pH 6.0 by 0.5 M ammonium carbonate. Afterwards, the produced suspensions were stored in a refrigerator overnight at 4 °C. Again, filtration was carried out, and the polysaccharide fucoidans were precipitated by the addition of three volumes of ethanol. To enhance fucoidans precipitation, the suspensions were stored again in the refrigerator at 4 °C overnight. Finally, the precipitates were recovered by centrifugation at 4500 rpm for 10 min followed by drying at 55 °C. The final products were stored in plastic tubes at room temperature in a desiccator.

### 3.4. Chemical Characterization of Extracted Fucoidans

Dubois or phenol-sulfuric acid assay, toluidine blue assay, and elemental analysis (CHNS Analysis) were conducted for total carbohydrate content, fucoidan content, and sulfation degree determination, respectively [68].

#### 3.4.1. Sugar Content

With some modifications from the original assay developed by Dubois et al., glucose (0.025–0.5 mg/mL) was used as a standards sugar for determination of neutral sugar monomers composition, where 200 µL of each sample were pipetted into a 2-mL reaction vessel followed by equal volume of 5% (*w/v*) phenol, and then 1.0 mL concentrated sulfuric was added gently. After 10 min, the reaction vessels were shaken vigorously for 15 s, 30 min incubation at room temperature, and then the absorbance measurement was conducted at 490 nm [69] by UV/Vis-spectrometer (Cary 60 UV-Vis, Agilent Technologies, Santa Clara, CA, USA).

#### 3.4.2. Fucoidan Content

In addition, fucoidans from *F. vesiculosus* and *Undaria pinnatifida* (≥95% pure, Sigma Aldrich^®^, St. Louis, MO, USA) were used as reference standards and construction of a suitable calibration curve in the range of 0.0–2.25 mg/mL. Into 990 µL of 0.06 mM TB dissolved in 20 mM maleic acid (pH 1), 10 µL of each sample were added before absorbance recording at 632 nm [48]. The results of *L. saccharina* and *F. spiralis* were expressed as mg *F. vesiculosus* and *U. Pinnatifida* equivalent/mg fucoidan extract.

#### 3.4.3. Sulfation Degree

Moreover, elemental Vario Micro cube apparatus (Elementar Analysensysteme GmbH, Langenselbold, Germany) calibrated with sulfanilic acid was employed for elemental analysis. The degree of polymer sulfation was calculated with regard to the ratio between %C and %S according to the equation described by Zayed et al. [42].

#### 3.4.4. Molecular Weight Determination

Weight-average molecular weight (Mw) was determined based on gel permeation chromatography (GPC) for the four fucoidans following our previous protocol [41] with some modifications. Briefly, the HPLC system was composed of a GPC_MCX column 8 × 30 mm (PSS, Mainz, Germany) combined with Shodex^®^ RI-101 detector (Shimadzu Corporation, Kyoto, Japan), while the pump P 6.1 L with autosampler AS 6.1 L were from Azura HPLC analytical system (KNAUER Wissenschaftliche Geräte GmbH, Berlin, Germany). Isocratic elution by 0.05 M phosphate buffer at pH 9.1 for 20.0 min, and a flow rate of 1.0 mL.min^−1^ were applied. The calibration standards (Dextran for GPC 5.0–670.0 kDa, Sigma-Aldrich^®^, St. Louis, MO, USA) and samples after filtration with a nylon membrane filter (0.2 µm) were analyzed in a final concentration of 2.0 mg·mL^−1^ in eluent containing 1.0 mg.mL^−1^ ethylene glycol. The column temperature was adjusted at 25 °C and the injection volume was 10 µL.

#### 3.4.5. Monomeric Composition

Following Obluchinskaya et al. with some modifications [12], 10 mg of each fucoidan sample were hydrolyzed in 0.5 mL 2 M trifluoracetic acid at 100 °C for 3 h. Following that, the solutions were cooled in ice bath and then the pH was adjusted at 7.0 by 2 M NaOH. The analyzed samples were hydrolyzed fucoidans in addition to several neutral sugar monomers as glucose, fucose, galactose, mannose, and rhamnose. The same HPLC system used previously with molecular weight was used except that the column was replaced with Repro-Gel Ca^2+^ (Dr. Maisch HPLC GmbH, Ammerbuch, Germany) provided with Ca^2+^ pre-column (Phenomenex, Torrance, CA, USA). Double deionized water was used for isocratic elution, while column temperature, injection volume, flow rate, and time of analytical protocol were 80 °C, 20 µL, 0.5 mg.mL^−1^, and 30 min were applied, respectively.

#### 3.4.6. Interfering Co-Extracted Compounds

Since brown seaweeds are rich in phenolic compounds, i.e., phlorotannins, which may be co-extracted and consequently may interfere with investigated bioactivities [2]. The TPC was estimated in the different fucoidans extract based on Folin–Ciocâlteu phenol reagent following the protocol used by El-Hawary et al. [70] with few modifications and gallic acid (1–200 µg/mL) was a reference for quantification. Briefly, 200 µL of each fucoidans were incubated after addition of 1.0 mL of Folin–Ciocâlteu reagent for 5.0 min in a dark place. Then, 800 µL of sodium bicarbonate (7.5 mg/mL) were added before a re-incubation step for 30 min. Finally, the absorbance was recorded at 765 nm. The TPC was expressed as mg gallic acid equivalent/mg extract (µg GAE/mg extract).

Additionally, protein content in fucoidans extract was performed according to Bradford method [71]. The experiment was conducted in 96-well microplate, where bovine serum albumin (BSA) was prepared in in a concentration range of 0.005–0.5 mg/mL as a reference standard. The absorbance measurement was conducted by Viktor^®^ X3 microplate reader (2030 Multilabel Reader, Perkin Elmer, Waltham, MA, USA) at 595 nm.

#### 3.4.7. Structural Features

Furthermore, ATR-FT-IR was employed for structural characterization using Spectrum 100 FT-IR (Perkin Elmer, Waltham, MA, USA). The technique was applied to elucidate the important structural features of extracted fucoidans, especially that are related to sulfation pattern [41].

### 3.5. Cytotoxic Activity

#### 3.5.1. Cell Lines and Cell Culture

The two commercial cell lines MCF7 (ATCC^®^ HTB-22™) and Caco-2 (ATCC^®^ HTB-37™), extracted from breast and colorectal adenocarcinoma, respectively, were purchased from LGC Standards GmbH (Wesel, Germany). The primary fibroblasts were gained from foreskin samples from phimosis and circumci-sion patients provided by Olgahospital Children’s Clinic (Stuttgart, Germany) by the outgrowth method. The procedure was performed according to the ethics committee of the state medical chamber of Baden-Württemberg, Germany (F-2021-108).

Cell culture media (high glucose Dulbecco’s Modified Eagle medium (DMEM) and Roswell park Memorial Institute (RPMI 1640) medium), fetal calf serum (FCS), penicillin-streptomycin, _L_-glutamine, and trypsin-EDTA were purchased from Thermo Fisher Scientific Inc. DMEM supplemented with 10% (*v*/*v*) FCS, 2 mM L-glutamine and 100 U/mL Penicillin-Streptomycin was used for cultivation of Caco-2 cells and primary fibroblasts. MCF7 cells were cultivated with RPMI 1640 supplemented with 10% (*v*/*v*) FCS, 2 mM L-glutamine and 100 U/mL Penicillin-Streptomycin. All cells were grown as adherent monolayers at humidified atmosphere at 37 °C and 5% CO_2_. 1x trypsin-EDTA (0.05%/0.53 mM EDTA) was used for passaging of Caco-2 cells and primary fibroblasts, for passaging of MCF7 cells 1x trypsin was utilized.

#### 3.5.2. Cytotoxicity Investigations

The cytotoxicity of the four extracted fucoidans (*L. saccharina*_MAE, *F. spiralis*_MAE, *F. vesiculosus*_MAE, and *F. vesiculosus*_SE) was analyzed in concentrations ranging from 4.9 µg/mL to 2500 µg/mL utilizing the cancerous cell lines Caco-2 and MCF7 as well as primary fibroblasts gained from healthy tissue following the previous protocol of Zayed et al. [42].

Briefly, 5000 cells were seeded per well of a 96-well plate (Corning, Falcon^®^, Amsterdam, The Netherlands) by dispensing 100 µL of a 100,000 cells/mL cell solution. The total volume of 200 µL per well was reached by adding fucoidan dilutions and the drug control methotrexate (Sigma Aldrich^®^) in phosphate-buffered saline without magnesium and calcium (PBS, Fisher Scientific Inc). For positive controls sodium dodecyl sulfate (SDS, Sigma Aldrich^®^) in PBS as negative control 100 µL cell-specific media was dispensed. Afterwards, the 96-well plates were incubated for five days under standard conditions (37 °C, 5% CO_2_, humidified atmosphere).

After these five days of incubation, phase contrast images were captured with a microscope (IX81 with XM10 camera, Olympus, Tokyo, Japan) equipped with a 10x objective lens. Afterwards, the supernatant in the 96-well plates was aspirated and the wells were washed with 200 µL PBS and filled with 200 µL PBS containing 10 µg/mL fluorescein diacetate (FDA, Sigma Aldrich^®^). Fluorescence (relative fluorescence units) was measured at 485 nm (excitation) and 538 nm (emission) after 15 min incubation at room temperature.

The CC_50_ values were calculated as 50% fluorescence intensity compared to that for the negative control (100% fluorescence intensity) and positive control (0% fluorescence intensity). Therefore, two-fold serial dilutions of the four fucoidan fractions starting from a stock solution of 5 mg/mL in PBS were prepared. All results were based on three experiments and are presented as mean ± standard deviation. For statistical analysis, a two-side Student *t*-test was used. Statistical significance was considered at *p* < 0.05.

## 4. Conclusions and Future Perspectives

MAE of fucoidans from different brown algae (e.g., *F. vesiculosus*, *F. spiralis*, and *L. saccharina*) was presented. The current research provided new insights into less investigated brown algae extraction by MAE technique which compared with the commonly used solvent extraction of fucoidans from marine sources. Using the same extraction conditions, the chemical characterization revealed the heterogeneity of fucoidans among brown algae orders, i.e., *Fucales* and *Laminariales*, where the fucoidan derived from *L. saccharina* showed different characters regarding higher sugar content and lower fucoidan content, in addition to less sulfation degree and unique sulfation pattern. However, the MAE resulted in lower molecular weight products compared with classical solvent extraction and other green extraction methods as EAE. The pre-treatment steps were successful for removal of potential co-extracted bioactive compounds that possibly interfered with fucoidans bioassays. Additionally, the cytotoxic effect on cell lines from adenocarcinomas of the microwave-assisted extracted *F. vesiculosus* fucoidan was the highest and safest among investigated fucoidans. This superior activity is possibly attributed to its galactofucan skeleton which possessed superior bioactivities compared to other classes of fucoidans, however the phenolic content residuals could not be excluded. Further extraction optimization and structural elucidation should be addressed in further work using different extraction conditions to improve the yield, products quality of other brown algae, and the underlined mechanism of action. Moreover, products purification and structural elucidation by NMR are highly recommended furtherly for full characterization of the produced fucoidans.

## Figures and Tables

**Table 1:** Extraction yield (% *w*/*w*) and chemical characterization of fucoidans extracted from *F. vesiculosus* using microwave-assisted extraction (MAE) at five different conditions listed in Table 4, by varying the parameters such as biomass/solvent ratio, microwave power%, and extraction time. The values are expressed as a mean of two biological replicates ± standard deviation.

Condition ≠	Chemical Character			
	Extraction Yield (% *w*/*w*)	Fucoidan Content (mg/mg Extract)	Sugar Content (mg Glucose Equivalent/mg Extract)	Sulfate Content (mg/mg Extract)
1	6.61 ± 0.040	0.643 ± 0.03	0.181 ± 0.08	0.092 ± 0.030
2	4.56 ± 0.006	0.770 ± 0.05	0.159 ± 0.22	0.042 ± 0.004
3	0.86 ± 0.004	0.459 ± 0.03	0.110 ± 0.05	0.043 ± 0.001
4	1.26 ± 0.000	0.351 ± 0.05	0.084 ± 0.02	0.069 ± 0.002
5	12.25 ± 0.011	0.595 ± 0.07	0.166 ± 0.09	0.062 ± 0.016

**Table 2:** Chemical characterization of fucoidans extracted from different brown algae. Fucoidans derived from *L. saccharina* and *Fucus spiralis* were extracted by microwave-assisted (MAE), while *F. vesiculosus* was extracted by both MAE and solvent extraction (SE) methods. The values are expressed as a mean of two biological replicates ± standard deviation.

Fucoidan Type	Chemical Character					
	Sugar Content (mg Glucose Equivalent/mg Extract)	Fucoidan Content Based on TB Assay		Sulfation Degree	Total Phenolic Content (µg Gallic Acid Equivalent (GAE)/mg Extract)	Protein Content (µg Bovine Serum Albumin Equivalent/mg Extract)
		(mg *F. vesiculosus* Fucoidan Equivalent/mg Extract)	(mg *U. pinnatifida* Fucoidan Equivalent/mg Extract)			
*L. saccharina*_MAE	0.47 ± 0.015	0.09 ± 0.06	0.11 ± 0.047	0.13	<1.0 ± 0.00	<5.0 ± 0.00
*F. spiralis*_MAE	0.12 ± 0.029	0.52 ± 0.035	0.43 ± 0.026	0.73		
*F. vesiculosus*_MAE	0.13 ± 0.015	0.65 ± 0.12	--- *	0.75	1.64 ± 0.23	
*F. vesiculosus*_SE	0.21 [47]	0.73 [47]	--- *	0.67 [47]	<1.0 ± 0.00	

*: Not determined.

**Table 3:** Calculated half-maximal cytotoxic concentration (CC_50_) of the four investigated fucoidan fractions against primary fibroblasts gained from healthy tissue and against cancerous cell lines MCF7 and Caco-2. The values were calculated from the dose–response data of different fucoidan fractions in concentrations ranging from 4.9 µg/mL to 2500 µg/mL (see Figure 2). Significant differences of CC_50_ values are given in Tables S1 and S2 in Supplementary Information.

Fucoidan	CC_50_ [µg/mL]		
	Primary Fibroblasts	MCF7	Caco-2
*F. vesiculosus*_SE	>2500.0	1784.2 ± 33.6	>2500.0
*F. vesiculosus*_MAE	>2500.0	45.9 ± 22.0	265.0 ± 94.9
*F. spiralis*	483.1 ± 73.7	266.7 ± 64.9	1091.6 ± 61.9
*L. saccharina*	2008.4 ± 44.9	1758.0 ± 68.1	1959.9 ± 11.1

**Table 4:** Different fucoidans extraction conditions from *F. vesiculosus* brown alga using MAE by varying extraction parameters such as biomass/solvent ratio, power%, and extraction time in duplicates (*n* = 2).

Conditions	Biomass: Solvent Ratio (g:mL)	Power (W)	Time (min)
1	1:25	560	1.0
2	1:25	560	2.0
3	1:10	560	1.0
4	1:10	240	1.0
5	1:25	240	1.0

## Data Availability

Not applicable.

## Supplementary Materials

The following supporting information can be downloaded at: https://www.mdpi.com/article/10.3390/md21010048/s1, Figure S1: Monomeric composition chromatograms of the four investigated brown algae crude fucoidan extracts. Figure S2: Relative cell viability of primary fibroblast (light grey bars), Caco-2 (dark grey bars) and MCF7 (white bars) cells after incubation with control substances: 300 µM methotrexate, PBS, cell-specific media (negative control) and SDS (positive control). The relative cell viability was calculated relative to the untreated negative control, Figure S3: Dose–response curves of primary fibroblasts (A), MCF7 cell line (B) and Caco-2 cell line (C) treated with four different fucoidan fractions in concentrations ranging from 4.9 µg/mL to 2500 µg/mL. The relative cell viability was calculated relative to the untreated negative control, Figure S4: Phase contrast images of different cell types (Caco-2 (upper row), MCF7 (middle row), primary fibroblasts (lower row) after incubation with cell specific media (negative control), SDS (positive control), 300 µM methotrexate and PBS. The scale bar of the image in the left corner represents 100 µm and is representative for all images. Table S1. Entitled differences of the calculated half-maximal cytotoxic concentration (CC_50_). The CC_50_ values within one cell type were compared. Significances are tagged as follows: * = *p* < 0.05, n.s. = not significant (*p* > 0.05), n.d. = not determined (at least one CC_50_ amounted > 2500 µg/mL). Table S2. Entitled differences of the calculated half-maximal cytotoxic concentration (CC_50_). The CC_50_ values within one fucoidan fraction were compared. For the fucoidan *F. vesiculosus*_SE no statistical evaluation was possible, since CC_50_ values were determined only for MCF7. Significances are tagged as follows: * = *p* < 0.05, n.s. = not significant (*p* > 0.05), n.d. = not determined (at least one CC_50_ amounted >2500 µg/mL).
