# Supplementary material for: Characterization and Cytotoxic Activity of Microwave-Assisted Extracted Crude Fucoidans from Different Brown Seaweeds
Source: Mar Drugs. 2023 Jan 11;21(1):48. doi: 10.3390/md21010048 (PMC9863780; doi:10.3390/md21010048)
Supplement: Supplementary file 1 [file marinedrugs-21-00048-s001.zip › marinedrugs-2142070-supplementary.pdf]

Article

Characterization and cytotoxic activity of microwave-assisted extracted crude fucoidans from different brown seaweeds

Supplementary Figures

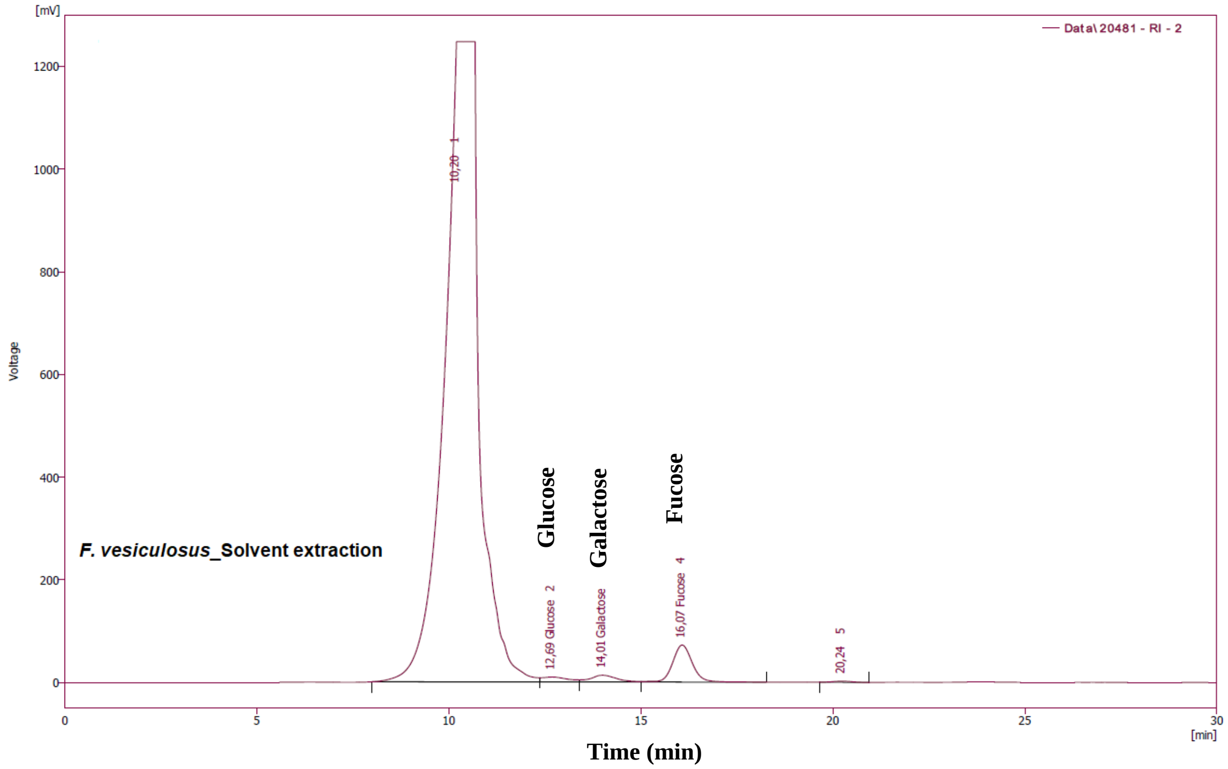

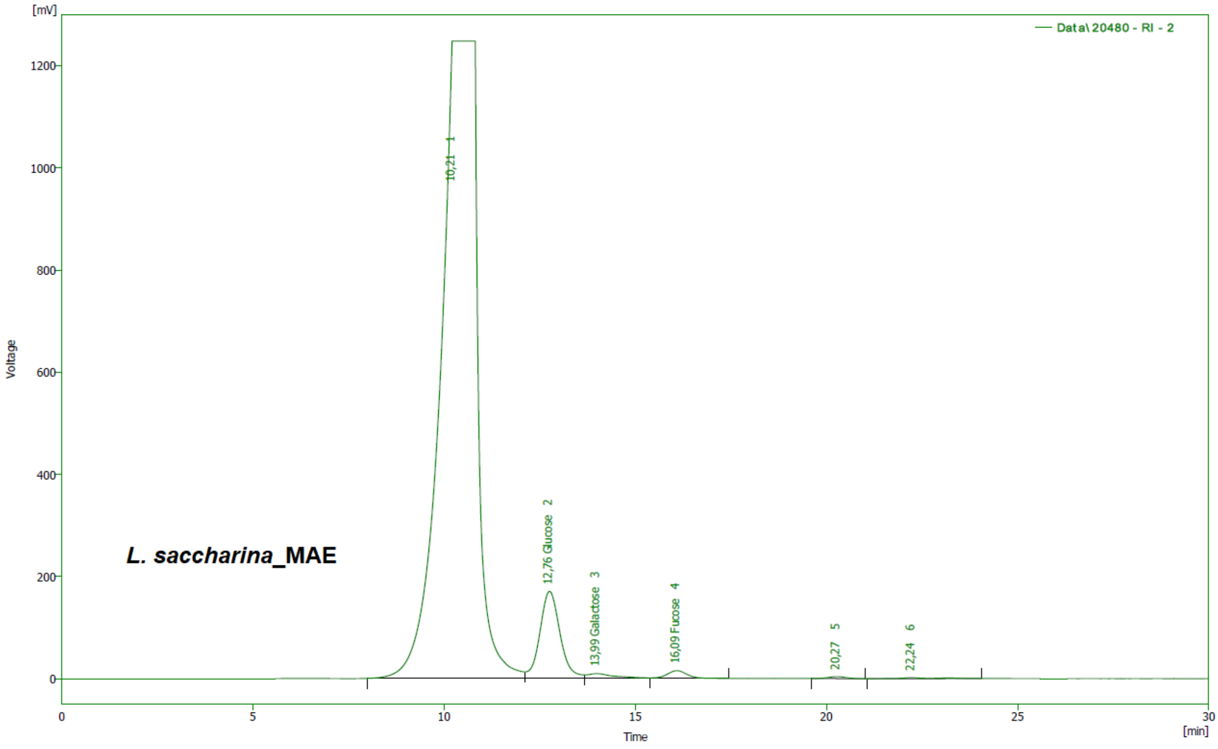

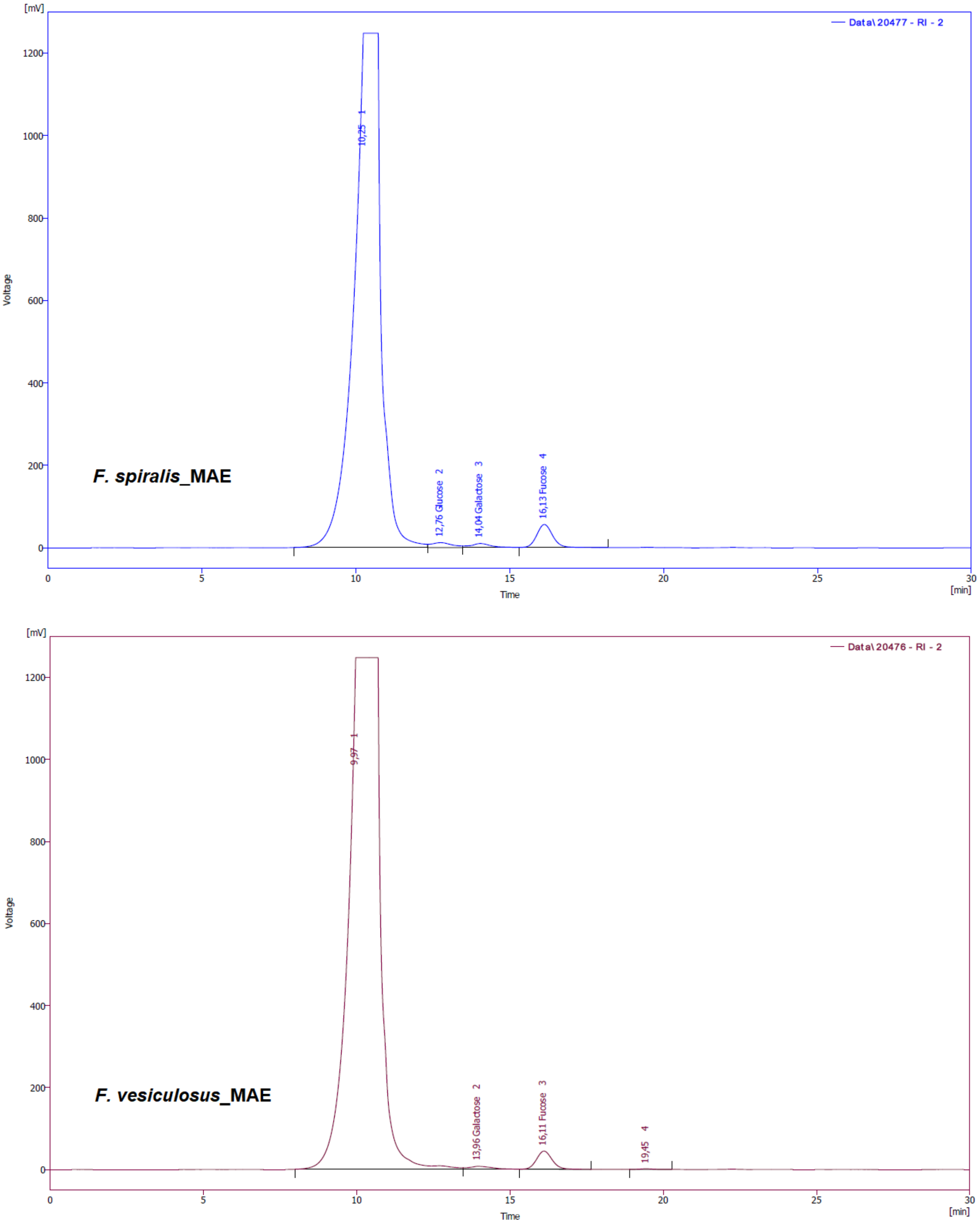

Figure S1: Monomeric composition chromatograms of the four investigated brown algae crude fucoidan extracts revealing the ratio between major sugar monomers, *i.e.*, glucose, galactose, and fucose.

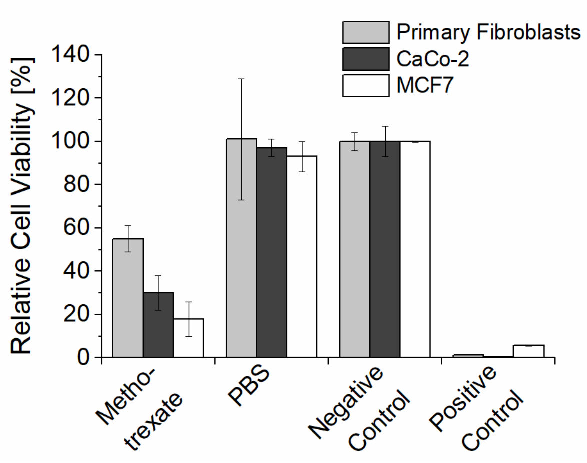

Figure S2: Relative cell viability of primary fibroblast (light grey bars), Caco-2 (dark grey bars) and MCF7 (white bars) cells after incubation with control substances: 300 µM methotrexate, PBS, cell-specific media (negative control) and SDS (positive control). The relative cell viability was calculated relative to the untreated negative control.

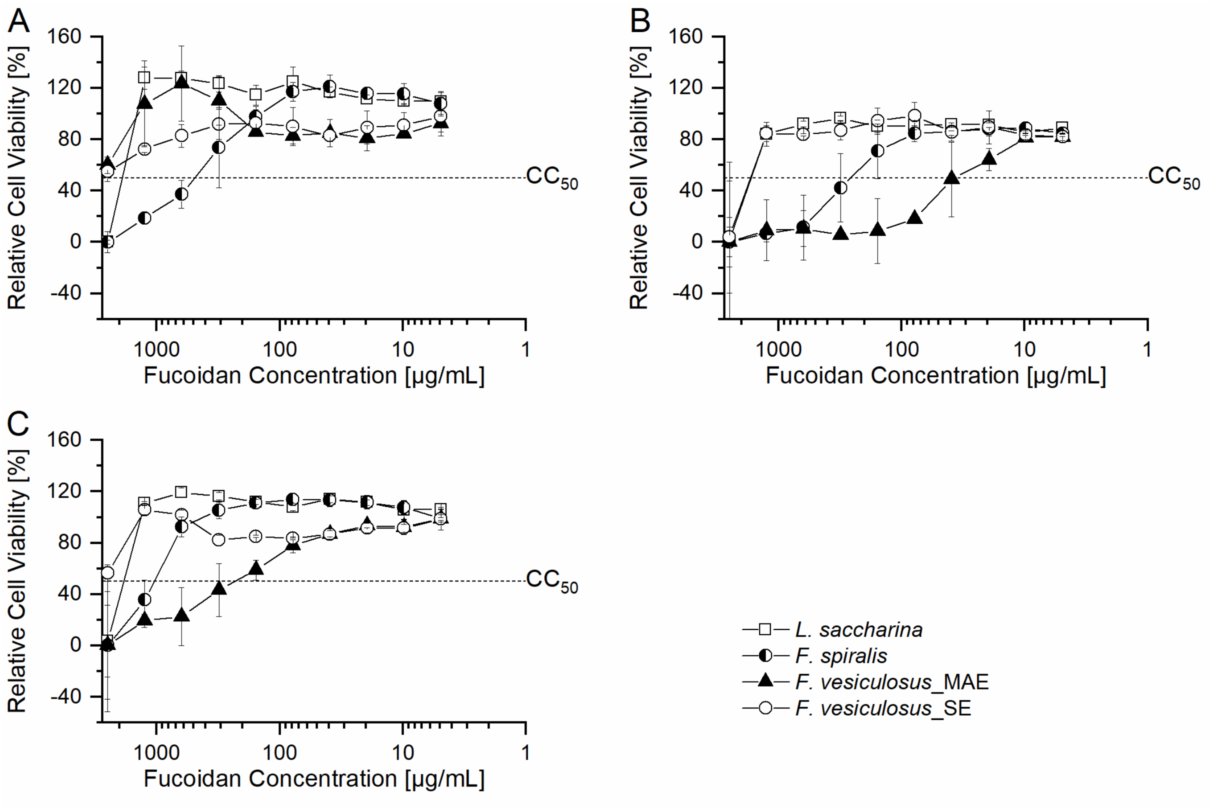

Figure S3: Dose-response curves of primary fibroblasts (**A**), MCF7 cell line (**B**) and Caco-2 cell line (**C**) treated with four different fucoidan fractions in concentrations ranging from 4.9 µg/mL to 2500 µg/mL. The relative cell viability was calculated relative to the untreated negative control.

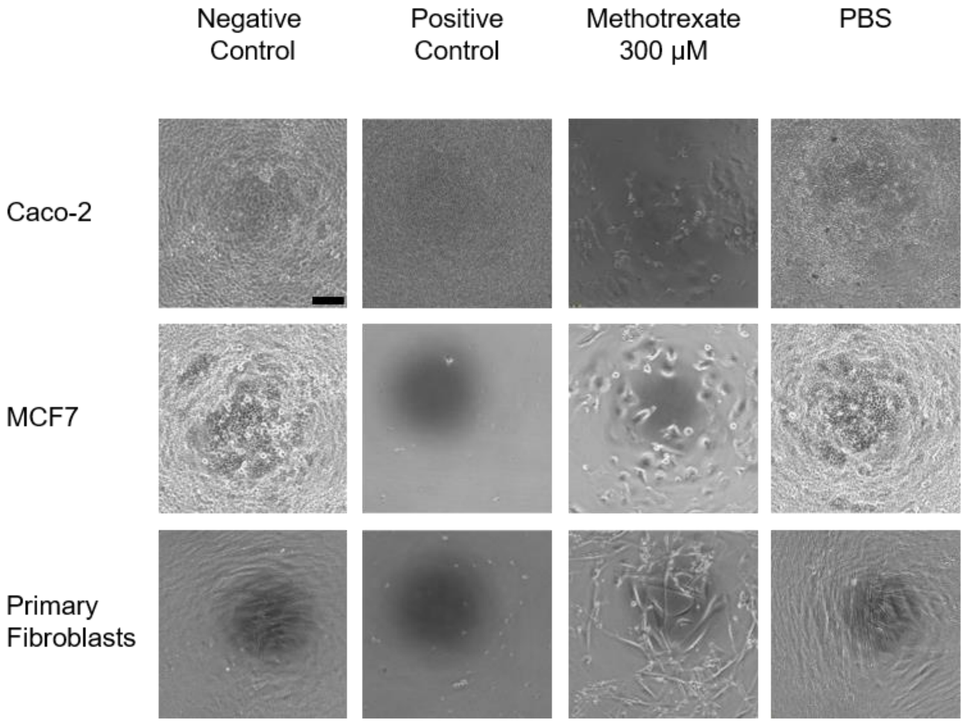

Figure S4: Phase contrast images of different cell types (Caco-2 (upper row), MCF7 (middle row), primary fibroblasts (lower row) after incubation with cell specific media (negative control), SDS (positive control), 300 µM methotrexate and PBS. The scale bar of the image in the left corner represents 100 µm and is representative for all images.

Supplementary Tables

Table S1. Entitled differences of the calculated half-maximal cytotoxic concentration (CC_{50}). The CC_{50} values within one cell type were compared. Significances are tagged as follows: * = p<0.05, n.s. = not significant (p>0.05), n.d. = not determined (at least one CC_{50} amounted >2500 µg/mL).

CaCo-2

	*F. vesiculosus*_SE	*F. vesiculosus*_MAE	*F. spiralis*	*L. saccharina*
*F. vesiculosus*_SE	-	n.d.	n.d.	n.d.
*F. vesiculosus*_MAE	n.d.	-	*	*
F. spiralis	n.d.	*	-	*
L. saccharina	n.d.	*	*	-

Primary Fibroblasts

	*F. vesiculosus*_SE	*F. vesiculosus*_MAE	*F. spiralis*	*L. saccharina*
*F. vesiculosus*_SE	-	n.d.	n.d.	n.d.
*F. vesiculosus*_MAE	n.d.	-	n.d.	n.d.
F. spiralis	n.d.	n.d.	-	*
L. saccharina	n.d.	n.d.	*	-

MCF7

	*F. vesiculosus*_SE	*F. vesiculosus*_MAE	*F. spiralis*	*L. saccharina*
*F. vesiculosus*_SE	-	*	*	n.s.
*F. vesiculosus*_MAE	*	-	*	*
F. spiralis	*	*	-	*
L. saccharina	n.s.	*	*	-

Table S2. Entitled differences of the calculated half-maximal cytotoxic concentration (CC_{50}). The CC_{50} values within one fucoidan fraction were compared. For the fucoidan *F. vesiculosus*_SE no statistical evaluation was possible, since CC_{50} values were determined only for MCF7. Significances are tagged as follows: * = p<0.05, n.s. = not significant (p>0.05), n.d. = not determined (at least one CC_{50} amounted >2500 µg/mL).

***F. vesiculosus*_MAE**

	Primary Fibroblasts	MCF7	Caco-2
Primary Fibroblasts	-	n.d.	n.d.
MCF7	n.d.	-	n.s.
Caco-2	n.d.	n.s.	-

F. spiralis

	Primary Fibroblasts	MCF7	Caco-2
Primary Fibroblasts	-	*	*
MCF7	*	-	*
Caco-2	*	*	-

L. saccharina

	Primary Fibroblasts	MCF7	Caco-2
Primary Fibroblasts	-	*	n.s.
MCF7	*	-	*
Caco-2	n.s.	*	-
